# Human Primary Epithelial Cells Acquire an Epithelial-Mesenchymal-Transition Phenotype during Long-Term Infection by the Oral Opportunistic Pathogen, *Porphyromonas gingivalis*

**DOI:** 10.3389/fcimb.2017.00493

**Published:** 2017-12-01

**Authors:** Jungnam Lee, JoAnn S. Roberts, Kalina R. Atanasova, Nityananda Chowdhury, Kyudong Han, Özlem Yilmaz

**Affiliations:** ^1^Division of Pulmonary, Critical Care and Sleep Medicine, University of Florida, Gainesville, FL, United States; ^2^Department of Oral Health Sciences, College of Dental Medicine, Medical University of South Carolina, Charleston, SC, United States; ^3^Department of Periodontology, University of Florida, Gainesville, FL, United States; ^4^Department of Nanobiomedical Science, BK21 PLUS NBM Global Research Center for Regenerative Medicine, Dankook University, Cheonan, South Korea; ^5^DKU-Theragen Institute for NGS Analysis, Cheonan, South Korea; ^6^Department of Microbiology and Immunology, Medical University of South Carolina, Charleston, SC, United States

**Keywords:** *Porphyromonas gingivalis*, oral opportunistic pathogen, oral epithelial cell, epithelial-mesenchymal-transition, oral cancer

## Abstract

*Porphyromonas gingivalis* is a host-adapted oral pathogen associated with chronic periodontitis that successfully survives and persists in the oral epithelium. Recent studies have positively correlated periodontitis with increased risk and severity of oral squamous cell carcinoma (OSCC). Intriguingly, the presence of *P. gingivalis* enhances tumorigenic properties independently of periodontitis and has therefore been proposed as a potential etiological agent for OSCC. However, the initial host molecular changes induced by *P. gingivalis* infection which promote predisposition to cancerous transformation through EMT (epithelial-mesenchymal-transition), has never been studied in human primary cells which more closely mimic the physiological state of cells *in vivo*. In this study, we examine for the first time in primary oral epithelial cells (OECs) the expression and activation of key EMT mediators during long-term *P. gingivalis* infection *in vitro*. We examined the inactive phosphorylated state of glycogen synthase kinase-3 beta (p-GSK3β) over 120 h *P. gingivalis* infection and found p-GSK3β, an important EMT regulator, significantly increases over the course of infection (*p* < 0.01). Furthermore, we examined the expression of EMT-associated transcription factors, Slug, Snail, and Zeb1 and found significant increases (*p* < 0.01) over long-term *P. gingivalis* infection in protein and mRNA expression. Additionally, the protein expression of mesenchymal intermediate filament, Vimentin, was substantially increased over 120 h of *P. gingivalis* infection. Analysis of adhesion molecule E-cadherin showed a significant decrease (*p* < 0.05) in expression and a loss of membrane localization along with β-catenin in OECs. Matrix metalloproteinases (MMPs) 2, 7, and 9 are all markedly increased with long-term *P. gingivalis* infection. Finally, migration of *P. gingivalis* infected cells was evaluated using scratch assay in which primary OEC monolayers were wounded and treated with proliferation inhibitor, Mitomycin C. The cellular movement was determined by microscopy. Results displayed *P. gingivalis* infection promoted cell migration which was slightly enhanced by co-infection with *Fusobacterium nucleatum*, another oral opportunistic pathogen. Therefore, this study demonstrates human primary OECs acquire initial molecular/cellular changes that are consistent with EMT induction during long-term infection by *P. gingivalis* and provides a critically novel framework for future mechanistic studies.

## Introduction

Oral cancer is the sixth most common cancer worldwide with an estimated 48,330 newly diagnosed cases and 9,570 deaths in 2016 (American Cancer Society, 2016). The incidence of oral cancer continues to increase in many parts of the world and occurs most frequently in men over the age of 50 (American Cancer Society, 2016). However, recent studies have shown that the incidence of oral cancer in people under 45 years is on the rise (Majchrzak et al., 2014), generating an even stronger and more urgent need for causal understanding and targeted therapeutic strategies. Various etiologic factors including alcohol use, smoking, and infective agents can trigger onset and accelerate progression of oral cancers (Atanasova and Yilmaz, 2014). On the other hand, the relationship between microbial infections and cancer as potential causative agents either directly or indirectly through enhanced inflammation, has been of recent interest in numerous studies (Atanasova and Yilmaz, 2014, 2015; Yuan et al., 2017). The opportunistic oral pathogen, *Porphyromonas gingivalis*, in particular, continues to gain momentum as potential risk modifier in the oral cancer research field (Atanasova and Yilmaz, 2015; Gao et al., 2016; Yuan et al., 2017).

*P. gingivalis* is a Gram-negative anaerobe and successful colonizer of oral epithelial cells (OECs), proposed as keystone pathogen primarily for its ability to promote a microbial environment favorable for disease (Hajishengallis et al., 2012; Spooner et al., 2016). In human OECs, *P. gingivalis* has multiple strategies by which it evades immune surveillance through the establishment of a replicative reservoir and the ability to spread to adjacent uninfected cells (Dorn et al., 2002; Yilmaz et al., 2006; Yilmaz, 2008; Hajishengallis, 2011; Choi et al., 2013; Hajishengallis and Lamont, 2014; Olsen and Hajishengallis, 2016). Once invaded, this opportunistic pathogen can manipulate the host machinery to facilitate its long-term survival by inhibiting the intrinsic apoptotic pathway (cytochrome c release and caspase 3/9 activation) (Yilmaz et al., 2004; Yao et al., 2010); modulating extracellular ATP-induced cellular reactive oxygen species and oxidative stress pathways (Yilmaz et al., 2008, 2010; Spooner and Yilmaz, 2011; Choi et al., 2013; Hung et al., 2013; Spooner et al., 2014; Johnson et al., 2015; Roberts et al., 2017); and attenuating pro-inflammatory cytokine IL-1β secretion and inflammasome pathways (Yilmaz et al., 2010; Choi et al., 2013; Hung et al., 2013; Johnson et al., 2015; Roberts and Yilmaz, 2015). In addition, live *P. gingivalis* promotes survival and proliferation of primary gingival epithelial cells through activation of the Phosphatidylinositol-4, 5-bisphosphate 3-kinase (PI3K)/protein-kinase B (Akt) pathway (Yilmaz et al., 2004; Yao et al., 2010) thereby preventing pro-apoptotic Bad activity and upregulation of cell cycle components (Kuboniwa et al., 2008; Pan et al., 2014). Therefore, these changes in the host signaling pathways due to *P. gingivalis* infection creates a unique environment for *P. gingivalis* to persist in the oral epi-mucosal tissues and thus be a major contributor to the progression of chronic periodontitis (Spooner et al., 2016). Intriguingly, epidemiological studies have found a significant relationship between periodontitis and oral squamous cell carcinoma (OSCC) (Costa et al., 2015; Galvao-Moreira and da Cruz, 2016; Cheng et al., 2017) and have also indicated the ability of *P. gingivalis* to enhance cancer mortality independent of periodontal disease (Ahn et al., 2012). Moreover, research shows a higher presence of *P. gingivalis* (33% higher) in gingival carcinomas than in normal gingiva (Katz et al., 2011). Accordingly, *P. gingivalis* has thus been proposed as a potential etiological agent to induce tumorigenesis and promote invasion of OSCC.

During EMT, epithelial cells lose their cell-cell adhesion and cell polarity but gain migratory and invasive properties (hallmarks of mesenchymal stem cells) (Larue and Bellacosa, 2005; Heerboth et al., 2015). Recent studies have shown that *P. gingivalis* infection enhances the aggressiveness, metastatic potential (Ha et al., 2015; Woo et al., 2017) and mortality (Ahn et al., 2012) of OSCC majorly through the induction of canonical EMT markers, matrix-metalloproteinases (MMP-9), β-catenin, zinc finger E-box-binding homeobox 1 (Zeb1) and vimentin, in immortalized oral epithelial cells (Zhou et al., 2015; Sztukowska et al., 2016). Furthermore, EMT changes, such as co-downregulation of E-cadherin and β-catenin, have a positive correlation with prognosis in OSCC (da Silva et al., 2015). Therefore, these recent studies collectively indicate that *P. gingivalis* infection may be a risk factor for OSCC (Geng et al., 2017) and may affect OSCC prognosis, aggressiveness and/or metastatic potential through the induction of EMT signaling. The use of immortalized cell lines in cancer research has been standard practice for decades and establishing a stable cell line from tumor cells has been an invaluable research tool for understanding progression and metastatic aspects of cancer biology (Binder Gallimidi et al., 2015). Similarly, the previous studies with *P. gingivalis* in this context were conducted using immortalized OECs either derived from cancer cell lines, transformed cells or TERT-immortalized keratinocytes, which all are shown to be predisposed to a higher risk of induction of malignant transformation with an enhanced proliferative phenotype as compared to primary cells (Alge et al., 2006; Hung et al., 2014; ATCC, 2016). In our study, we utilize human primary OECs to investigate the early molecular changes of EMT induced during *P. gingivalis* infection which provides us with a different validation approach and a model more closely mimics the physiological state of human cells in *vivo* prior to transformation.

Therefore, to explore the potential effect of long-term *P. gingivalis* infection on initial EMT changes and elucidate an EMT signaling mechanism in human primary OECs, we studied 72–120 h of infection (3–5 days) and examined the expression and localization of canonical EMT markers. The results demonstrate that *P. gingivalis* infection inhibits glycogen-synthase kinase-3 beta (GSK3β), thus leading to EMT shifts resulting in the reduced expression of E-cadherin and membrane-associated β-catenin. This is coupled with the increased expression of transcriptional repressors Slug and Snail in primary human OECs, as well as increased Vimentin and MMPs expression which are all well-established to promote an invasive and metastatic phenotype in various cancers (Kessenbrock et al., 2010; Liu et al., 2010; Satelli and Li, 2011; Kidd et al., 2014; Basu et al., 2015).

Therefore, we establish a molecular signaling pathway important for *P. gingivalis* associated EMT changes in the presence of long-term infection in human primary OECs, which lays a biologically relevant foundation for future mechanistic studies and may lead to the identification of future therapeutic targets. Furthermore, these results support the putative role of *P. gingivalis* as a risk modifier in OSCCs through the induction of EMT signaling.

## Materials and methods

### Primary cell culture

Primary human oral epithelial cells (OECs) were obtained from adult patients who were selected randomly and anonymously from those presenting for tooth crown lengthening or impacted third molar extraction. Gingival tissue was collected under the approved guidance of the University of Florida Health Science Center Institutional Review Board (IRB, human subjects assurance number FWA 00005790). No patient information was collected and the informed consent was obtained by all subjects. The OECs were cultured in serum-free keratinocyte growth medium (KGM, Lonza) at 37°C in 5% CO_2_ as previously described (Lamont et al., 1995; Yilmaz et al., 2003, 2008). After seeding into the culture flask, cells were not passaged for the duration of the respective infection hours described in later methods. The OECs are viable and attached for the duration of the experimental times with and without infection (up to 120 h) (Supplementary Figure 1) and were also shown to be viable and metabolically active with the DMSO treatment alone (Supplementary Figure 3).

### Bacterial infection

*P. gingivalis* ATCC 33277 was cultured anaerobically at 37°C in Trypticase soy broth (TSB) supplemented with yeast extract (1 μg/mL), hemin (5 μg/mL), and menadione (1 μg/mL). This strain is found present in human OSCC samples (Katz et al., 2011; Sztukowska et al., 2016) as well as in individuals with high risk for the oro-digestive cancer—pancreatic cancer (Michaud et al., 2013). The bacteria were cultured overnight and harvested in mid-log phase by centrifugation at 6,000 g for 10 min at 4°C. The bacterial pellets were re-suspended in sterile Dulbecco's phosphate-buffered saline (PBS) containing calcium and magnesium (HyClone), and bacteria were quantified using a Klett-Summerson photometer. *Fusobacterium nucleatum* ATCC 25586 was cultured anaerobically at 37°C in Brain–Heart Infusion broth supplemented with yeast extract (5 mg/mL), hemin (5 μg/mL), and menadione (1 mg/mL). Bacteria were harvested in mid-log phase. OECs at approximately 60% confluence were infected with the bacteria at a multiplicity of infection (MOI) of 100 in all experiments. It is important to note that an inoculum of MOI 100 has been consistently shown to have the optimal attachment and invasion rate in primary OECs as compared to other MOI's such as MOI 10, and was therefore used throughout this study (Lamont et al., 1995; Yilmaz et al., 2002, 2004, 2006, 2008; Choi et al., 2011). Furthermore, this inoculum does not induce detachment of the primary OECs from the substratum in our primary cell model (Supplementary Figure 1). We also demonstrate that *P. gingivalis* is viable and metabolically active during 120 h of infection (Supplementary Figure 2).

### Western blot analyses

Western blot analyses were used to evaluate the level of EMT markers (GSK3β, Slug, E-cadherin, MMP7, Vimentin) in OECs infected with *P. gingivalis* for 72, 96, and 120 h. Infections were performed backwards and thus all cells including uninfected and 120 h-infected cells were examined at the same stage of growth to diminish any effects of cell aging on the protein expression. Total proteins were extracted from the infected OECs using RIPA lysis buffer plus protease and phosphatase inhibitors: 1 mM PMSF; 0.1 mM TLCK; 1 mM NaF; 2 mM N-ethylmaleimide; 1 mM sodium orthovanadate; and aprotinin (10 μg/ml). Protein concentration of each sample was measured using Bradford assay (BioRad) to determine equal amounts (30 ~ 50 μg) of protein samples were loaded onto either a 12 or 15% SDS polyacrylamide gel. Cell supernatants were concentrated using Trichloroacetic acid (TCA) protein precipitation (Link and LaBaer, 2011). For secreted proteins, a common method for normalizing blots is to analyze both supernatants and cell lysates from the same population, and use a reference protein from the cell lysates to show the secreted proteins are being detected from approximately the similar number of cells. β-tubulin was used as a reference protein from cell lysates in the same population. After gel electrophoresis, the proteins were transferred onto a nitrocellulose membrane using wet-transfer system and the membrane was blocked in Tris-buffered saline with 0.1% Tween 20 (TBST) containing 5% nonfat dry milk. The membrane was incubated overnight at 4°C with anti-EMT marker rabbit monoclonal antibodies E-cadherin, vimentin, slug, and phosphoGSK3β (Cell signaling); MMP-7 antibody (Santa Cruz) at a dilution rate of 1:1,000 in TBST. Horseradish peroxidase conjugated anti-rabbit antibody (Cell signaling) was used for secondary labeling at 1:1,000 in TBST for 1 h at room temperature. Protein bands were visualized using enhanced chemiluminescence (ECL, GE Healthcare). The membrane was re-probed with β-tubulin mouse antibody (Invitrogen) at 1:1,000 in TBST. A horseradish peroxidase conjugated anti-mouse (Cell Signaling) was used to visualize anti-β-tubulin. Protein band intensities were examined using NIH ImageJ.

### Immunofluorescence microscopy

To examine β-catenin distribution, OECs were seeded on glass coverslips (Warner Instruments) in four-well plates (ThermoFisher Scientific) and infected at an MOI 100 with *P. gingivalis* 33277 for 120 h. At 120 h post-infection, the infected OECs were fixed with 4% paraformaldehyde for 30 min and permeabilized for 30 min in PBS containing 0.1% Triton X-100. The permeabilized cells were incubated with rabbit anti- β-catenin monoclonal antibody (Cell signaling) at 1:100 dilution and mouse anti- *P. gingivalis* 33277 polyclonal antibody at 1:1000 in PBS containing 0.1% Tween 20 for 1 h. After washing with PBS-Tween 20, cells were immunostained with Alexa Fluor 568 goat anti-mouse immunoglobulin G (Invitrogen) at 1:500 dilution and Alexa Fluor 488 goat anti-rabbit immunoglobulin G (Invitrogen) at 1:1,000 dilution at ambient temperature for 1 h. The immunostained cells were mounted on glass slides using Vectashield mounting media with DAPI, and examined using wide-field fluorescence microscope (Zeiss Axio Imager A1). The images were captured using a cooled charge-coupled device camera controlled by QCAPTURE software (Qimaging). To examine E-cadherin in uninfected or *P. gingivalis*–infected OECs, cells were fixed with10% neutral buffered formalin for 30 min and permeabilized for 30 min in PBS containing 0.1% Triton X-100. The permeabilized cells were incubated with rabbit anti- E-cadherin monoclonal antibody (Cell signaling) at 1:250 dilution and Alexa Fluor 488 goat anti-rabbit immunoglobulin G (Invitrogen) at 1:1,000 dilution.

### Zymography

MMP2 and MMP9 activations were examined in supernatants harvested from uninfected or *P. gingivalis*-infected OECs culture using gelatin zymography. Protein samples were precipitated from the cell culture supernatant using TCA precipitation method (Link and LaBaer, 2011) and loaded on 10% SDS-polyacrylamide gel containing 3% gelatin. The gels were incubated at ambient temperature with renaturing buffer (Novex) two times for 30 min each, followed by overnight incubation at 37°C with developing buffer (Novex). After staining with Coomassie Brilliant Blue R-250, enzymatic activities of MMP2 and MMP9 were visualized as clear bands against a dark blue background.

### Sybr green quantitative RT-PCR

Transcription of *Snail* and *Zeb1* were measured by quantitative real time PCR, using the Sybr Green detection system. The following pairs of primers were used: human-specific *Snail* (5′CACTATGCCGCGCTCTTT3′forward; 5′TGCTGGAAGGTAAACTCTGGAT3′ reverse), human-specific *Zeb1* (5′CACATGCGATTACATTCTGGAG3′ forward; 5′CGTGCTCATTCGAGAGGATT3′reverse), and human GAPDH-specific (5′GAAATCCCATCACCATCTTCCAGG3′ forward; 5′GAGCCCCAGCCTTCTCCATG3′ reverse). GAPDH was used as an internal control. Total RNA was extracted from uninfected or *P. gingivalis*-infected primary OECs with RNeasy Plus Mini Kit (Qiagen), and 1 μg of the total RNA per sample was reverse-transcribed using a High Capacity cDNA Reverse Transcription Kit (Applied Biosystems). Quantitative PCR was conducted at 95°C for 3 min, followed by 45 cycles at 95°C for 30 sec and either 57°C (for *Snail* and *Zeb1*) or 62°C (for GAPDH) for 30 sec. Gene expression analysis was performed using the CFX Manager Software (BioRad). The measured expression of GADPH was used as a reference gene. For each condition, uninfected OECs were assigned as control. The control quantity, which is set to 1, was used by the software to further normalize the relative quantities for *Zeb1*.

### Scratch migration assay

OECs were seeded in 12-well plates and infected with an MOI 100 of either *P. gingivalis* 33277 or *F. nucleatum* 25586 for 72 h. Cell culture media was changed at 24 h intervals. After 72 h of infection a scratch was made in each well using a pipette tip. Cells were monitored for migration in fresh media containing the proliferation inhibitor, Mitomycin C (5 μg/mL). Migrating cells were monitored at 12 h using Zeiss Invertoskop 40C Microscope. The images were captured using a cooled charge-coupled device camera controlled by QCAPTURE software (Qimaging).

### Zone of exclusion migration assay

A sterile glass cylinder (Bioptech) was placed in the center of each well in a 6-well plate. OECs were seeded in the 6-well plates outside of the glass cylinder thus creating a Zone of Exclusion (Nyegaard et al., 2016). The OECs were then infected with an MOI 100 of either *P. gingivalis* 33277 and/or *F. nucleatum* 25586 for 72 h. The glass cylinder was then removed and cells were monitored at 12 h for migration in fresh media containing the proliferation inhibitor, Mitomycin C (5 μg/mL) using Zeiss Invertoskop 40C Microscope. The images were captured using a cooled charge-coupled device camera controlled by QCAPTURE software (Qimaging).

### Statistical analysis

The expression of EMT markers in *P. gingivalis*-infected OECs were compared to those of uninfected OECs unless otherwise specified. The assessment was evaluated by two-tailed Student's *t*-test. *P*-values of 0.05 or less were considered to be statistically significant.

## Results

### *P. gingivalis* increases phosphorylation of GSK3β in OECs over the course of infection

**Figure 1:**
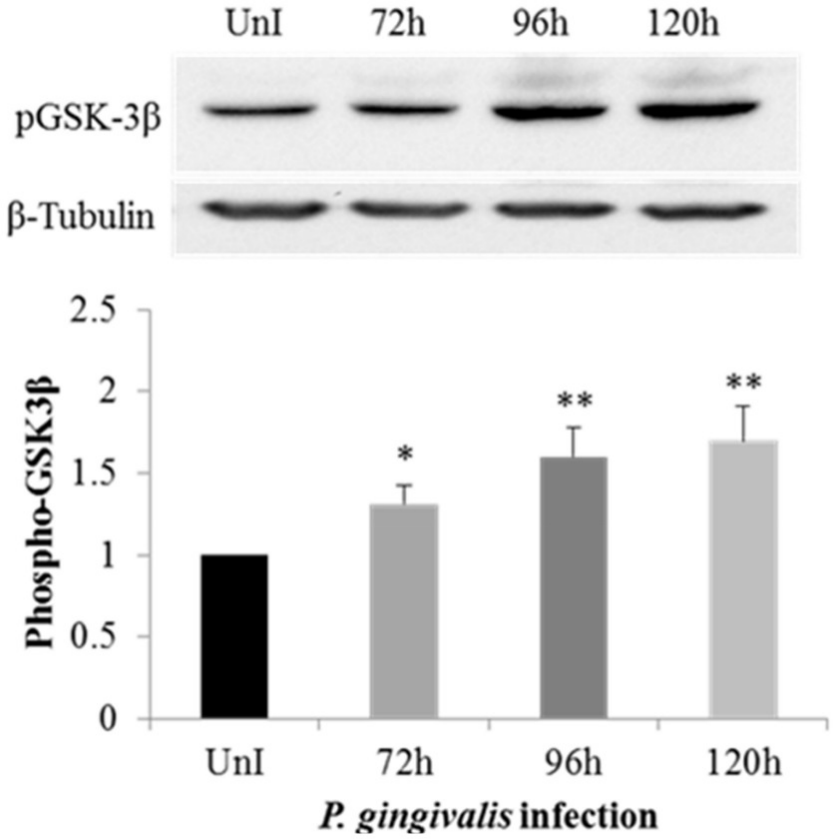
Phosphorylation of GSK3β is increased in primary OECs by *P. gingivalis* infection. OECs were either uninfected or incubated with *P. gingivalis* at an MOI of 100 for 72, 96, and 120 h. UnI denotes uninfected OECs. Cell lysates were collected and analyzed by immunoblot with a GSK3β phospho-specific antibody. The absolute intensities of phosphorylated GSK3β bands were measured using ImageJ software and the relative intensities were calculated using β-tubulin as a loading control. Data are shown as the mean and standard deviation of three independent experiments. ^*^Denotes statistical significance *p* < 0.05 and ^**^Denotes statistical significance *p* < 0.01 as compared to uninfected according to two-tailed Student's *t*-test.

Previously, we have shown that *P. gingivalis* inhibits host cell apoptosis through activation of the PI3K pathway (Yilmaz et al., 2004) which also has critical regulatory roles in EMT-related changes in cancer (Larue and Bellacosa, 2005). One well-explored path is the phosphorylation of Glycogen synthase kinase 3 beta (GSK3β), on serine 9 leading to its inactivation (Fang et al., 2000). The phosphorylation of GSK3β has been explored in many cancers as an initiator for promoting EMT phenotypes (Mishra, 2010; McCubrey et al., 2014; Mishra et al., 2015). Therefore, we speculated that *P. gingivalis* infection may induce the phosphorylation of GSK3β in primary OECs. We examined the level of phosphorylated GSK3β in *P. gingivalis*-infected OECs at 72, 96, and 120 h-post-infection, and compared them with uninfected OECs. The level of phosphorylated GSK3β is significantly increased over the course of *P. gingivalis* infection (Figure 1).

### Snail and slug EMT-inducing transcription factors are significantly increased in *P. gingivalis*-infected OECs

**Figure 2:**
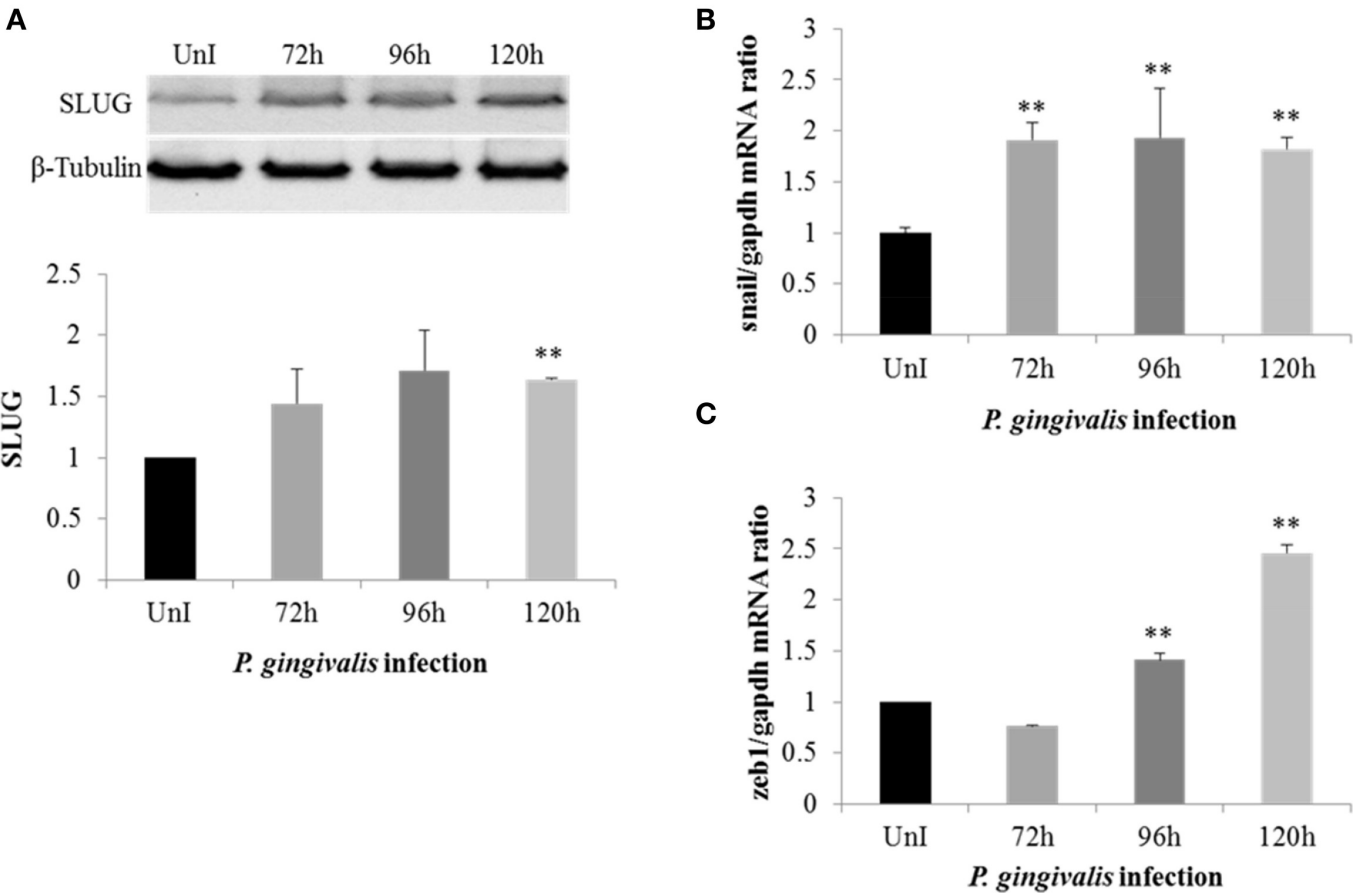
*P. gingivalis* increases expression of key EMT-promoting transcription factors (Slug, Snail, and Zeb1) in primary OECs. OECs were incubated with *P. gingivalis* at an MOI of 100 for 72, 96, and 120 h and compared to an uninfected control. **(A)** Cell lysates were extracted and immunoblotted with a Slug antibody. The absolute intensities of Slug bands were measured using ImageJ software and the relative intensities were calculated using β-tubulin as a loading control. **(B,C)** SybrGreen detection of mRNA expression levels of *Snail* or *Zeb1* using qRT-PCR. The data is represented as the mean and standard deviation of three independent experiments. ^**^Denotes statistical significance (*p* < 0.01) as compared to uninfected according to two-tailed Student's *t*-test.

To further investigate the effect of *P. gingivalis* in inducing EMT in primary OECs we analyzed the expression of Snail and Slug which are EMT-inducing transcription factors normally degraded by GSK3β (Zhou et al., 2004; Bachelder et al., 2005; Kao et al., 2014). Since long-term *P. gingivalis* infection increases the inhibitory phosphorylation of GSK3β, it is reasonable to assume there would be a corresponding increase in Snail and Slug expression in *P. gingivalis*-infected OECs. Our results show the protein expression of Slug was significantly increased in *P. gingivalis*-infected OECs (Figure 2A). Moreover, the expression of *Snail* was also significantly increased in *P. gingivalis* infected cells at 72 h-post-infection and remained consistently increased over 120 h of *P. gingivalis* infection (Figure 2B). We also analyzed the expression of *Zeb1*, a marker of EMT that is positively correlated with the expression of *Snail* (Guaita et al., 2002). Analysis of *Zeb1* mRNA levels in *P. gingivalis* infected and uninfected OECs (Figure 2C) reveal significantly increased expression in *P. gingivalis*-infected OECs beginning at 96 h-post-infection. The sequential increase of *Snail* expression starting at 72 h post-infection followed by *Zeb1* at 96 h is consistent with literature which demonstrates Snail expression precedes Zeb1expression in EMT (Guaita et al., 2002).

### Loss of E-cadherin expression leads to altered β-catenin distribution in *P. gingivalis*-infected OECs

**Figure 3:**
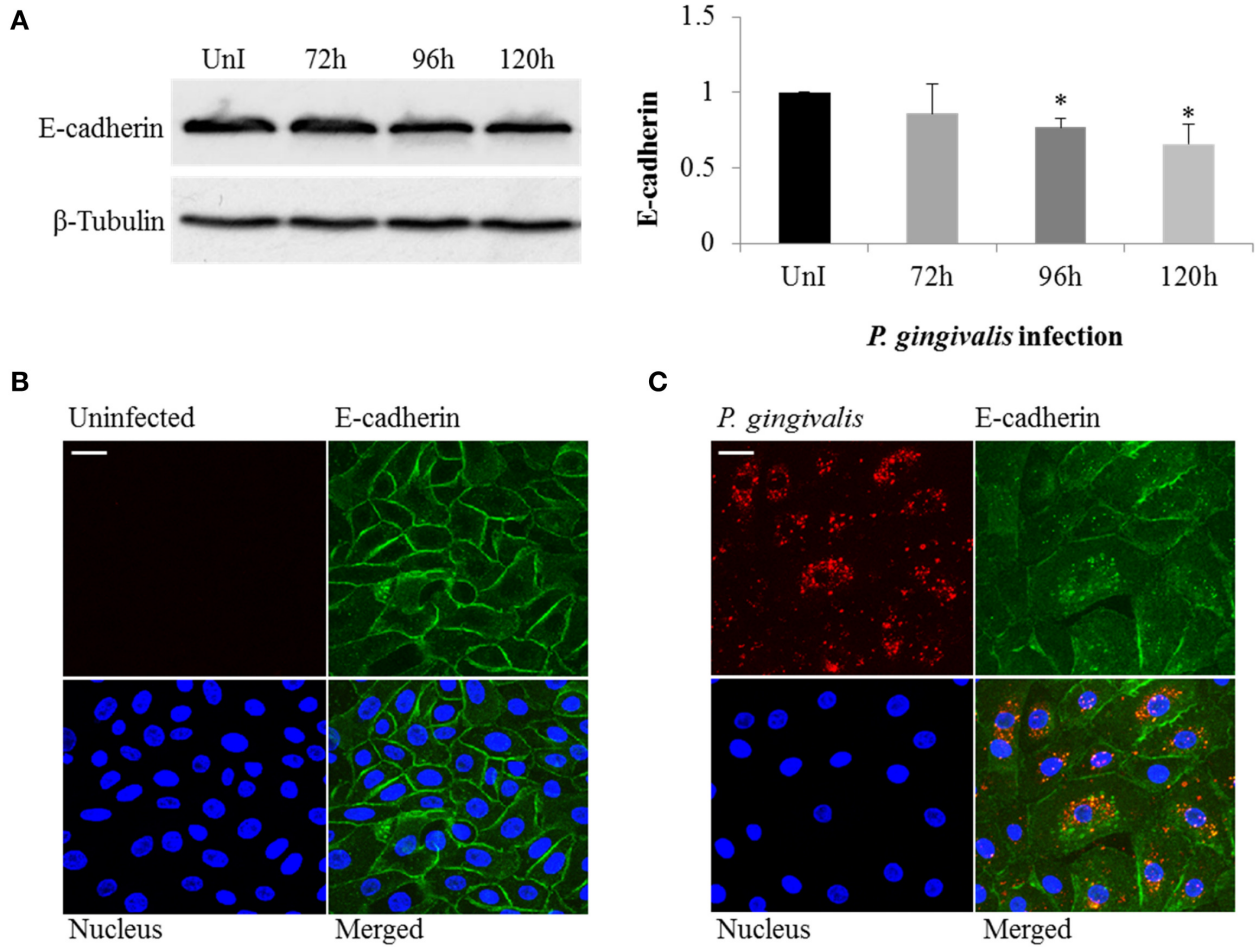
*P. gingivalis* induces loss of E-cadherin in infected primary OECs. OECs were incubated with *P. gingivalis* at an MOI of 100 for 72, 96, and 120 h and compared to an uninfected control. **(A)** Cell lysates were extracted and immunoblotted with an E-cadherin antibody. The absolute intensities of E-cadherin bands were measured using ImageJ software and the relative intensities were calculated using β-tubulin as a loading control. **(B,C)** Micrographs of OECs infected with *P. gingivalis* for 120 h imaged on Zeiss Axio imager at 40X magnification. Uninfected and *P. gingivalis*-infected cells were fixed, permeabilized, and immunostained with a monoclonal E-cadherin antibody (green) and an antibody for *P. gingivalis* (red). Nuclei were stained with DAPI (blue). At least three separate fields containing an average of 20 OECs were studied in each of the three independent experiments performed in duplicate. Bar 10 μm.

**Figure 4:**
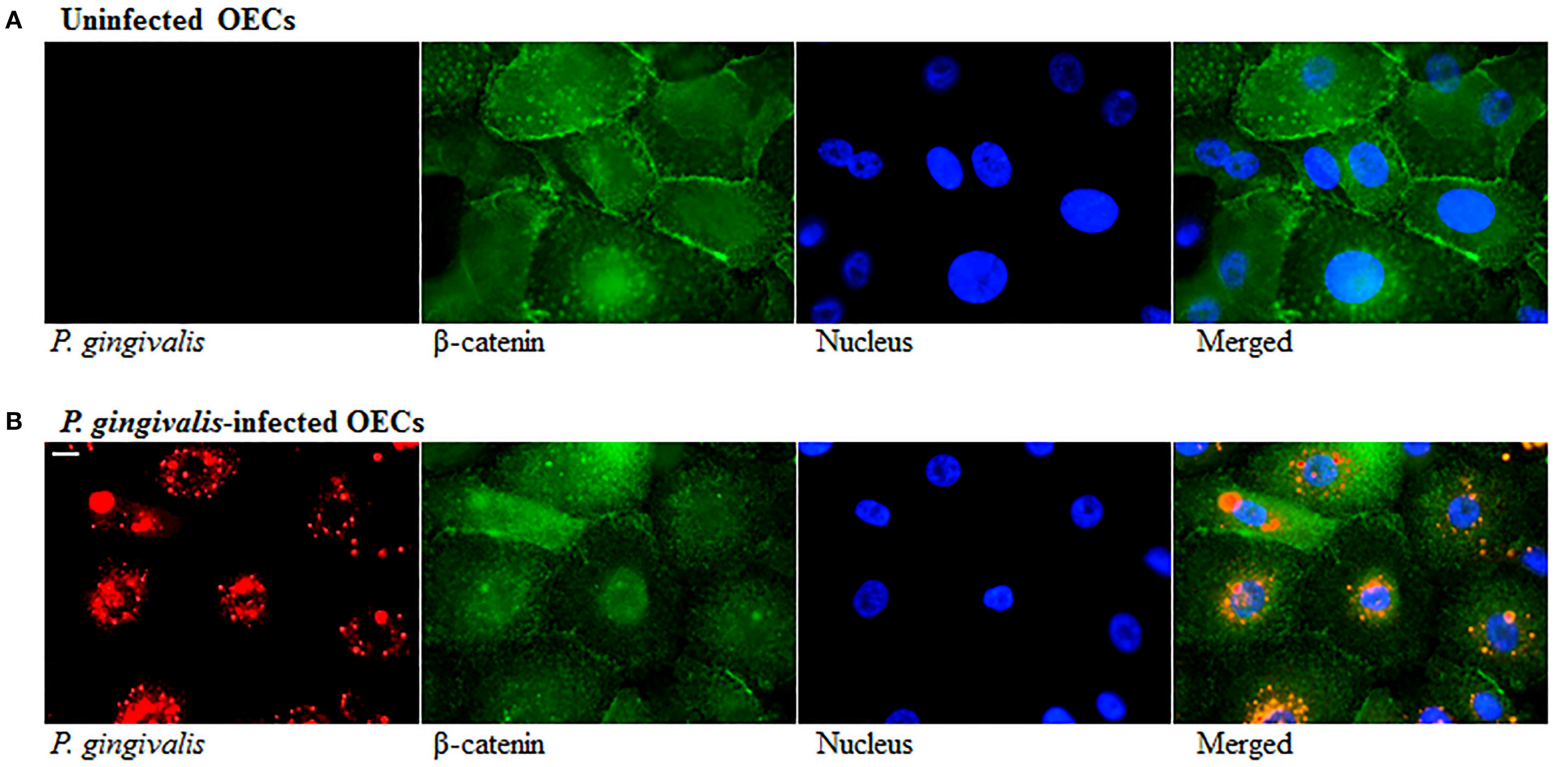
Altered Distribution of β-Catenin in *P. gingivalis*-infected cells. OECs were incubated with *P. gingivalis* at an MOI of 100 for 120 h. **(A)** Uninfected OECs. **(B)** *P. gingivalis*-infected OECs. Uninfected and *P. gingivalis*-infected cells were fixed, permeabilized, and immunostained with a monoclonal β-catenin antibody (green fluorescence) and for a rabbit *P. gingivalis* antibody (red-orange fluorescence). Nuclei were stained with DAPI (blue fluorescence). At least three separate fields containing an average of 20 OECs were studied in each of the three independent experiments performed in duplicate. Bar 10 μm.

The loss of E-cadherin, an integral membrane protein essential for epithelial architecture and attachment, due to increased Snail and Slug expression in cancers is well characterized. Furthermore, loss of E-cadherin is one of the most common biological indicators for EMT (Krisanaprakornkit and Iamaroon, 2012; Costa et al., 2015). Therefore, we examined E-cadherin in *P. gingivalis*-infected and uninfected OECs and found E-cadherin protein expression markedly decreased in OECs over 120 h of *P. gingivalis* infection (Figure 3A). Micrographs of immunostained OECs at 120 h post-infection also confirm a marked loss of E-cadherin on the membrane (Figure 3B). In normal epithelial cells, β-catenin binds to the cytoplasmic tail of E-cadherin and is sequestered on the membrane, therefore loss of E-cadherin expression results in the release of β-catenin into the cytoplasm leading to enhanced tumor progression (Heuberger and Birchmeier, 2010; Costa et al., 2015). Moreover, *P. gingivalis* has also been shown to activate β-catenin independent of host signaling pathways through proteolytic processing by gingipains (Zhou et al., 2015). We therefore examined the distribution of β-catenin in uninfected and 120 h *P. gingivalis*-infected OECs using immunostaining (Figure 4). Micrographs depict a shift in the distribution of β-catenin from the cell peripheries in uninfected OECs to a concentrated appearance in the nucleo-cytoplasmic subcellular region of *P. gingivalis* infected OECs.

### *P. gingivalis* promotes expression of matrix metalloproteinases and vimentin

**Figure 5:**
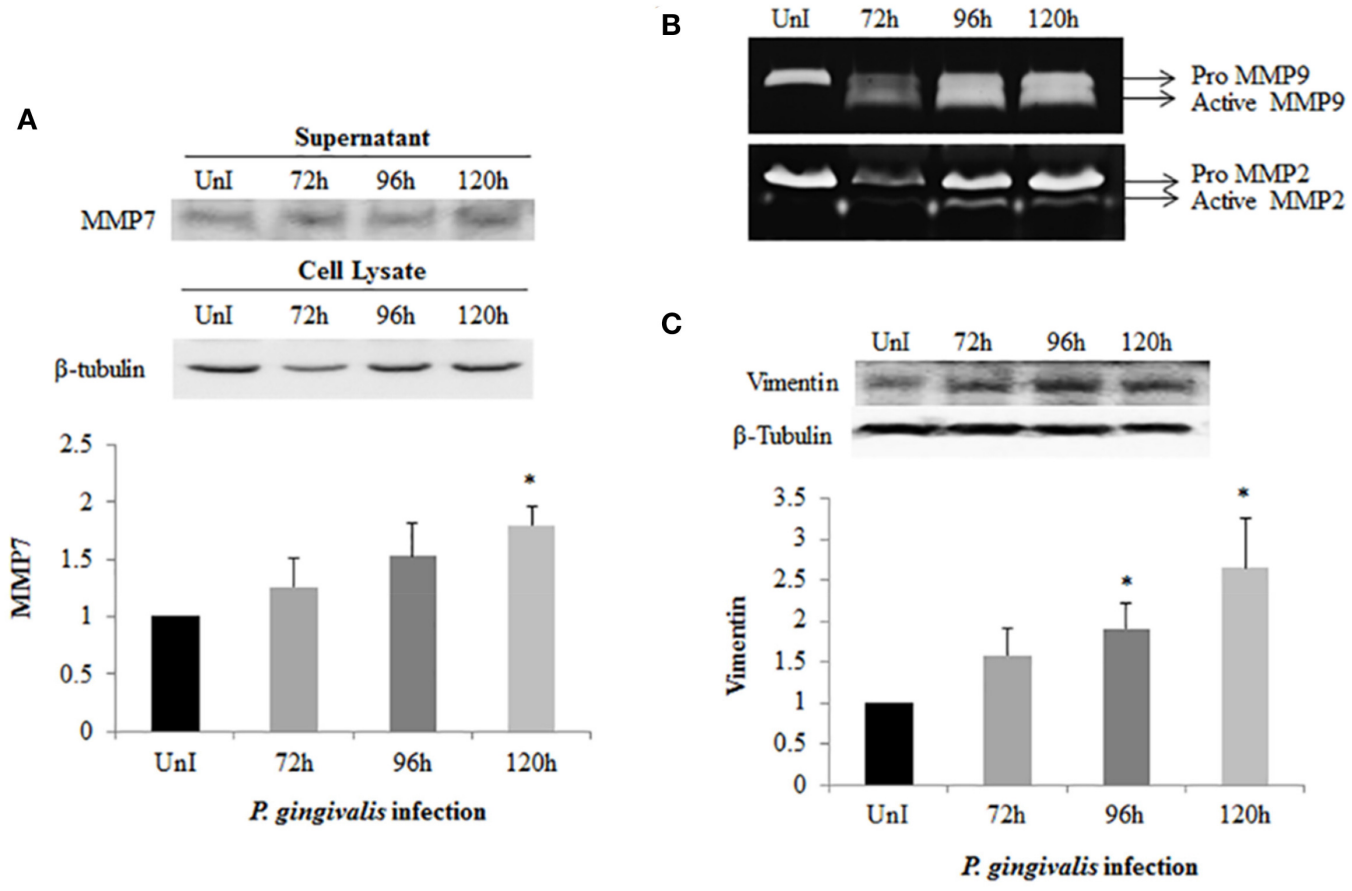
Activation of MMPs and Vimentin expression in *P. gingivalis*-infected OECs. OECs were incubated with *P. gingivalis* at an MOI of 100 for 72, 96, and 120 h. Bradford assay was used to determine the protein concentration of each sample. **(A)** Supernatants were harvested and precipitated using the TCA method from uninfected and *P. gingivalis*-infected OECs. The precipitated proteins were immunoblotted and detected using a monoclonal MMP7 antibody. **(B)** TCA-precipitated proteins were loaded on a SDS-PAGE gel containing 3% of gelatin. The gelatinolytic activity of MMP2 and MMP9 were visualized as clear, non-staining regions of the gel. **(C)** Cell lysates were extracted from uninfected and *P. gingivalis*-infected OECs and immunoblotted with a monoclonal Vimentin antibody. The absolute intensities of MMP7 and Vimentin western blot bands were measured using ImageJ software and the relative (normalized) intensities were calculated using β-tubulin as a loading control. Data are shown as the mean ± standard deviation of three independent experiments. ^*^Denotes statistical significance (*p* < 0.05) according to two-tailed Student's *t*-test.

Matrix metalloproteinases (MMPs) are calcium-dependent zinc-containing endopeptidases which degrade components of the extracellular matrix (ECM). During EMT, MMPs are upregulated and their activities correlate with tumor cell invasion, motility, and metastasis (Hong et al., 2000; Qiao et al., 2010; Krisanaprakornkit and Iamaroon, 2012). Furthermore, the regulation of MMP expression has been associated with increased β-catenin, Snail, and Slug expression. Therefore, we harvested and precipitated supernatants from uninfected and *P. gingivalis*-infected cells to examine the presence of secreted MMP7 (Figure 5A). Western blot analysis demonstrates a steady significant increase in secreted MMP7 over 120 h of *P. gingivalis* infection. We also examined MMP2 and MMP9 expression using gelatin zymography and found that *P. gingivalis* infection also promotes MMP2 and MMP9 activity in primary human OECs (Figure 5B). This data is consistent with other data which shows increased expression of MMPs in the presence of *P. gingivalis* infection (Fravalo et al., 1996; Zhou and Windsor, 2006; Atanasova and Yilmaz, 2014), which suggests that *P. gingivalis* promotes motile and invasive characteristics of OECs.

**Figure 6:**
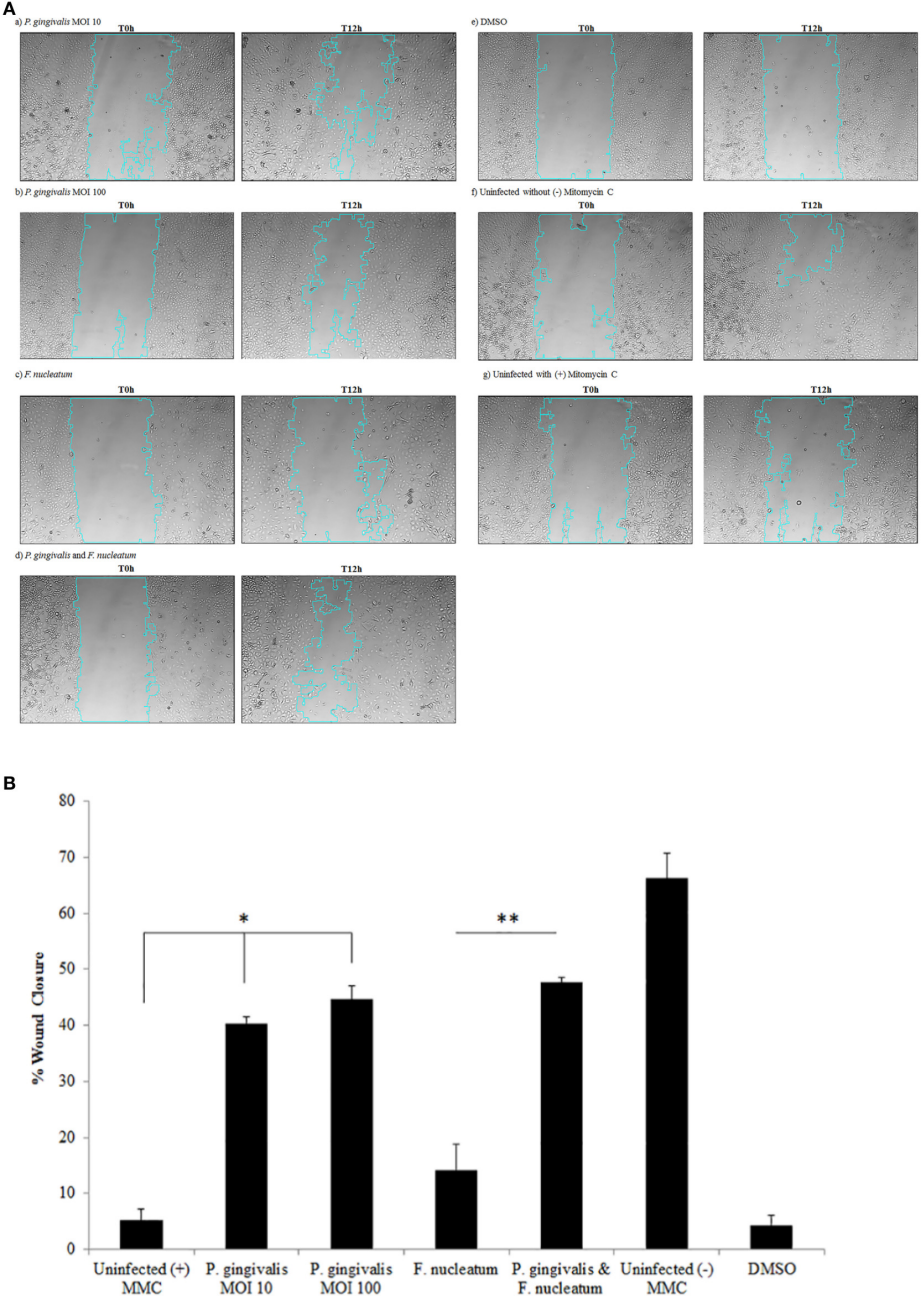
*P. gingivalis* infected cells promote migration of OECs over 120 h as determined by a Wound Healing/Scratch Migration Assay. Primary OECs were infected with *P. gingivalis* 33277 MOI 10 or MOI 100 and/or *F. nucleatum* 25586 at MOI 100. **(A)** The scratch wound was made 72 h post-infection and OECS were treated with Mitomycin C (5 μg/mL). Representative micrographs shown are of OECs at 0 h post-wound and 12 h post-wound: (a) *P. gingivalis* MOI 10; (b) *P. gingivalis* MOI 100; (c) *F. nucleatum*; (d) *P. gingivalis*, and *F. nucleatum*; (e) DMSO 0.1%; (f) Uninfected without (–) Mitomycin C; (g) Uninfected with (+) Mitomycin C. **(B)** The NIH ImageJ Wound Healing Tool was used to analyze the area of Wound Closure in primary OECs infected with *P. gingivalis* 33277 MOI 10 or MOI 100 and/or *Fusobacterium nucleatum* 25586 at MOI 100. The scratch wound was made 72 h post-infection and OECS were treated with Mitomycin C (5 μg/mL). Student *T*-test: ^*^p < 0.01 as compared to Uninfected with (+) Mitomycin C; ^**^p < 0.01 as compared to *F. nucleatum* infected. The data is represented as the mean ± SEM; *n* = 3.

**Figure 7:**
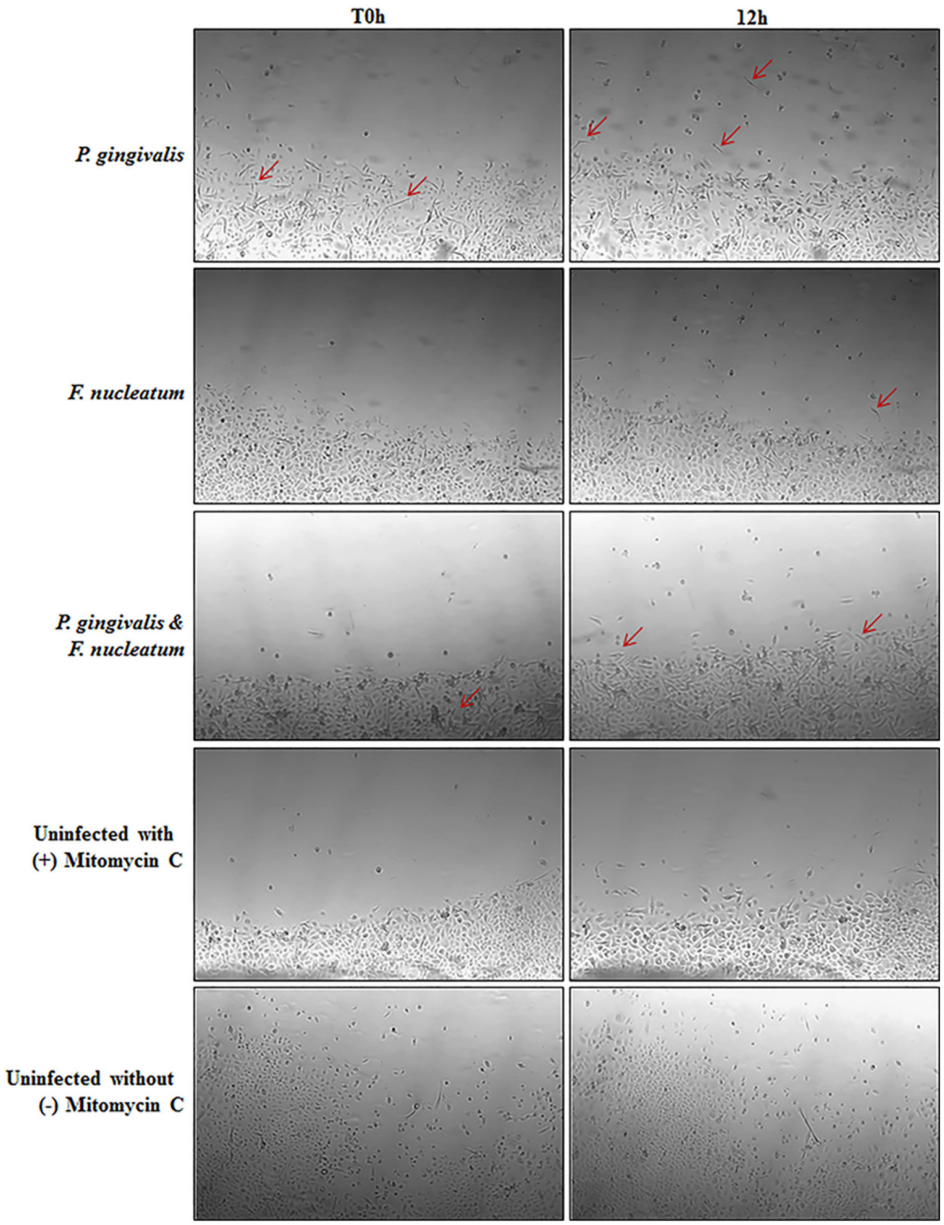
Micrographs depicting morphological changes in *P. gingivalis* infected cells as compared to Untreated in a Zone Inhibition Migration Assay. Primary OECs were infected with *P. gingivalis* 33277 and/or *F. nucleatum* 25586 at an MOI 100. OECS were then treated with Mitomycin C (5 μg/mL) to inhibit proliferation. Glass cylinders creating an obstructed zone in the well were removed at 72 h post-infection. Representative micrographs shown are of OECs at 72 h post-infection (T0 h) and 12 h after removal of the glass cylinder. Red arrows highlight select cells with a mesenchymal phenotype.

Vimentin, an intermediate filament expressed in mesenchymal cells, is a canonical marker for EMT which also promotes cell motility and an invasive phenotype (Kidd et al., 2014). In this way, it has been suggested that vimentin may be a marker for the metastatic potential of epithelial-derived tumors (Satelli and Li, 2011; Kidd et al., 2014). Examination of Vimentin protein expression in uninfected and *P. gingivalis*-infected OECs showed that the level of Vimentin is significantly increased by *P. gingivalis* infection (Figure 5C). We further examined the effects of these early molecular EMT changes on the migration potential of primary OECs using both a standard Wound Healing/Scratch assay (Liang et al., 2007) and a Zone of Exclusion Migration Assay (Nyegaard et al., 2016). The migration assay results reveal that long-term *P. gingivalis* infection promotes primary OEC migration, with an MOI of 100 having a slightly higher percent wound closure in our system than an MOI of 10. Further, co-infection with *P. gingivalis* and *F. nucleatum*, another oral opportunistic pathogen, displays a slight increase in wound closure compared to *P. gingivalis* infection alone (Figure 6). Moreover, primary OECs infected with *P. gingivalis* present more mesenchymal cell morphology as compared to untreated cells or cells infected with *F. nucleatum* (Figure 7); whereas, the migration assays indicate a rounded and dispersed morphology of the primary OECs in the presence of *F. nucleatum* infection, which may be indicative of a more invasive phenotype. This is consistent with a recent *in vivo* oncogenesis study which elegantly shows *F. nucleatum* can play a potential role in cancer cell invasiveness and suggests microbial synergy with *P. gingivalis* (Rubinstein et al., 2013; Atanasova and Yilmaz, 2014; Binder Gallimidi et al., 2015; Holt and Cochrane, 2017).

**Figure 8:**
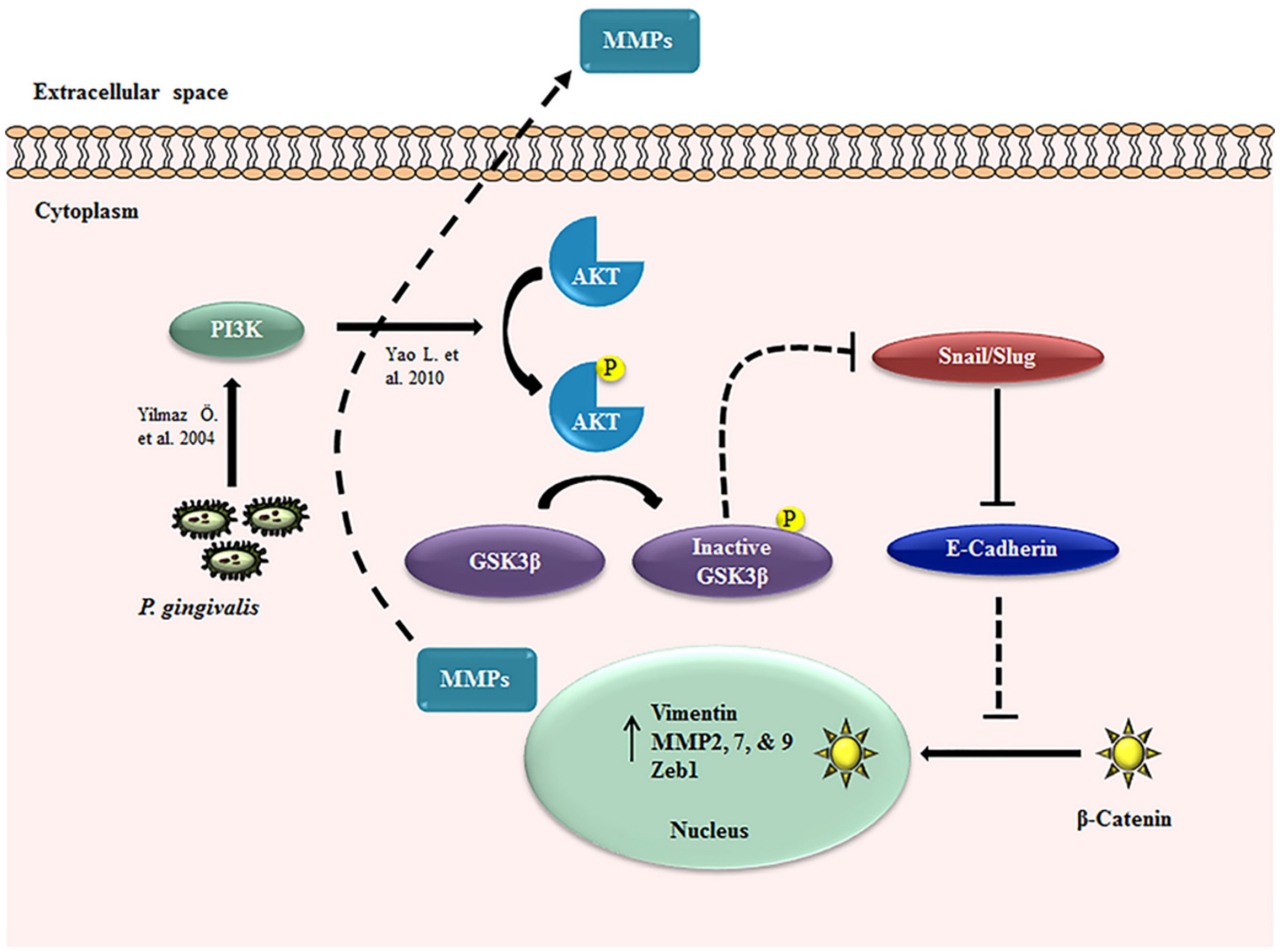
EMT signaling induced during *P. gingivalis* infection in primary OECs. Schematic diagram of the proposed EMT signaling mechanisms in OECs during long-term infection by *P. gingivalis*. The dotted line indicates the pathway without *P. gingivalis* infection and the solid lines represent the path in the presence of infection: *P. gingivalis* increases PI3K/Akt activation (Yilmaz et al., 2004; Yao et al., 2010) which can lead to the inactivation of GSK3β, resulting in the increased expression of Snail and Slug transcription factors. These molecular changes promote the loss of E-cadherin and nucleo-cytoplasmic accumulation of β-catenin. The three transcription factors, Snail, Slug, and β-catenin induce the increased expressions of Zeb1, Vimentin, and MMP2, 7, and 9.

Taken together these data suggest that human primary OECs display significant increases in the expression of MMPs and Vimentin which are largely associated with metastatic potential in cancers (Kessenbrock et al., 2010; Liu et al., 2010; Satelli and Li, 2011; Kidd et al., 2014; Basu et al., 2015) and promote a migratory phenotype in the host cells during extended period of infection by *P. gingival* is (Figure 8).

## Discussion

Epithelial-mesenchymal transition (EMT) is a normal biological process in development and also a necessary component for wound healing at later stages in life (Voulgari and Pintzas, 2009; Heerboth et al., 2015). The process of EMT involves the loss of cell-cell adhesion in epithelial cells and cell polarity complemented with the gain of migratory and invasive properties (hallmarks of mesenchymal stem cells) (Chaw et al., 2012; Krisanaprakornkit and Iamaroon, 2012; Costa et al., 2015). In addition, EMT is also investigated to be linked to cancer initiation, invasion and metastasis (Krisanaprakornkit and Iamaroon, 2012; da Silva et al., 2015; Heerboth et al., 2015). Moreover, EMT-related changes are found early in the development of OSCC and have been linked to poor prognosis in oral squamous cell carcinomas (OSCC) (Krisanaprakornkit and Iamaroon, 2012; da Silva et al., 2015). Investigations into the critical signaling pathways which lead to EMT involved in OSCC identify the activation of the PI3K/Akt signaling pathway as a recurrent event (Hong et al., 2009; Iamaroon and Krisanaprakornkit, 2009). Studies further reveal, using constitutively active Akt models, that this pathway promotes EMT in OSCC through downregulation of E-cadherin (a cell adhesion molecule), β-catenin (a subunit of the cadherin complex and transcription signal inducer), and upregulation of Vimentin (a mesenchymal intermediate filament) (Satelli and Li, 2011). These events led to morphological changes that increased cell motility and invasiveness (Hong et al., 2009). Further understanding of the GSK3β pathway which induces EMT identifies additional components critical in EMT induction. This includes transcription factors Snail, Slug and Zeb1. Multiple studies show the function of GSK3β in the degradation of Snail and Slug (Zhou et al., 2004; Bachelder et al., 2005; Wang et al., 2013; Kao et al., 2014); therefore the inhibition of GSK3β leads to the increased expression of these transcription factors. The main function of the two transcription factors is to suppress the expression of E-cadherin (Yokoyama et al., 2001). In addition, the expression of Zeb1 is increased along with Snail in tumor cells undergoing EMT (Guaita et al., 2002). This signaling cascade and upregulation of transcription factors, set into motion by the phosphorylation of GSK3β (Fang et al., 2000), leads to loss of E-cadherin, β-catenin, increased Vimentin and matrix metalloproteinase (MMP) expression.

E-cadherin is expressed in most epithelial cells and functions in maintaining the normal structure of tissues as well a cell polarity (Liu et al., 2010; Chaw et al., 2012). It is a transmembrane glycoprotein which interacts with catenins such as β-catenin to promote cell-adhesion and connection with the actin cytoskeleton (Berx and van Roy, 2009). The loss of E-cadherin expression is well established as an EMT marker in epithelial cells jointly with increased expression of Vimentin (Christiansen and Rajasekaran, 2006; Liu et al., 2010; Chaw et al., 2012). β-catenin is part of the complex that connects E-cadherin to the actin cytoskeleton and plays an important role in EMT onset and progression regulated in part by GSK3β (Clevers, 2006). In the absence of GSK3β, β-catenin is not targeted for degradation and can translocate to the nucleus and bind lymphoid-enhancing factor/T-cell factor (LEF/TCF), to initiate the transcription of target genes important for EMT-changes and cell proliferation (Gilles et al., 2003). MMP7 is a direct transcriptional target of β-catenin signaling and is regulated by β-catenin in human colorectal cancer, pancreatic cancer, and breast cancer (Brabletz et al., 1999; Crawford et al., 1999; Li et al., 2005). Other targets include Vimentin and MMPs 2 and 9 (Gilles et al., 2003; Wu et al., 2007). We show in primary OECs, the loss of E-cadherin and β-catenin from the cell peripheries, and nucleo-cytoplasmic distribution of β-catenin during the infection. The subcellular localization of β-catenin we observed here is consistent with other β-catenin regulation studies (Krieghoff et al., 2006). The visualized nucleo-cytoplasmic accumulation may result from either the loss of E-cadherin or from proteolytic processing of the degradation complex housing β-catenin in the cytoplasm (Zhou et al., 2015). In the referenced study, the non-canonical activation of β-catenin by *P. gingivalis* still led to nucleo-cytoplasmic translocation and increased activity of the TCF/LEF promoter in the immortalized TIGK cells (Zhou et al., 2015). Therefore, the loss of E-cadherin and the non-canonical activation of β-catenin may both be important during EMT induced by *P. gingivalis* infection, thereby allowing for β-catenin to potentially participate in transcriptional activation. However, the investigation of the alleged proteolytic processing was not in scope of this study. Nonetheless, the increase in the specific transcriptionally regulated downstream targets of β-catenin described above in our study, suggests that β-catenin is active in human primary OECs infected by *P. gingivalis*.

Vimentin is the main intermediate filament in mesenchymal calls and it not expressed in normal epithelial cells (Satelli and Li, 2011; Kidd et al., 2014). It is largely associated with an invasive phenotype in tumors and is believed to be in part regulated by β-catenin (Chaw et al., 2012). Matrix metalloproteinases (MMPs) play an important role in tumor invasiveness and vary in expression in different cancer types (Kessenbrock et al., 2010). The roles of MMPs include effects on cell apoptosis, growth signals, migration, and extracellular matrix regulation and even tumor vasculature (Kessenbrock et al., 2010). MMP7 is increased in many malignant cancer types and is secreted specifically from epithelial cells (Basu et al., 2015). The MMPs 2 and 9 correlate with increased metastatic potential and are found at higher levels in OSCCs (Hong et al., 2000).

In the induction of these described EMT-signaling pathways and mesenchymal phenotypic changes, microbial pathogens have been shown to play an important role as initiating factors (Atanasova and Yilmaz, 2014). For example, well-host adapted opportunistic organism, *Helicobacter pylori* induces EMT through MAPK/Erk-NF-kB signaling pathway and suppresses GSK3β activity by stimulating the PI3K/Akt pathway in gastric cancer (Polk and Peek, 2010; Wroblewski et al., 2010). Interestingly, it is shown that intracellular presence of *H. pylori* can play a vital role in the induction of gastric diseases including stomach cancer when the intracellular survival also facilitates the microorganism's persistence in the gastric mucosa (Dubois, 2007). In this study, we demonstrate that *P. gingivalis*, an opportunistic facultative intracellular pathogen's long-term infection in OECs strongly associates with the induction of early EMT-changes as summarized in Figure 8. It is logical to consider that the early molecular and cellular EMT events induced may originate from the sustained activation of PI3K/Akt pathway which has previously been shown to be markedly increased by *P. gingivalis* at 24 h post-infection in primary OECs (Yilmaz et al., 2004; Yao et al., 2010). Other studies later have shown *P. gingivalis* can selectively activate the PI3K/Akt pathway through either lipopolysaccharide -LPS- (Martin et al., 2003) or fimbriae (Yilmaz et al., 2002, 2004; Hajishengallis et al., 2009) acting on Toll-like receptor 2/β-integrin pathways (Hajishengallis et al., 2006).

The early molecular changes explored in this study, however, begin with an observed increase in GSK3β phosphorylation, which promotes increased expression of Snail, Slug, and Zeb1 transcription factors. Consequently, there was loss of E-cadherin coupled with accumulation and altered localization of β-catenin inside the primary human oral epithelial cells (OECs). In addition to the loss of E-cadherin we observed the increased expression of Vimentin and MMPs 2, 7, and 9 which are shown to be transcriptionally regulated by β-catenin (Brabletz et al., 1999; Gilles et al., 2003; Li et al., 2005). The culmination of these molecular events appears to promote an enhanced migratory and mesenchymal phenotype in primary OECs, as shown in our migration assays. Together these results suggest the ability of *P. gingivalis* to induce initial early EMT events in epithelial cells which may enhance tumor progression and invasion. This is supported by several studies which investigate the mechanisms of *P. gingivalis*' promotion of tumorigenic properties in the oral cavity using various immortalized cell lines as models and find similar increased expression of EMT markers such as Zeb1 and MMPs (Fravalo et al., 1996; Katz et al., 2011; Sztukowska et al., 2016; Geng et al., 2017). It is important to note that *P. gingivalis* may also work synergistically with other periodontal bacteria such as *F. nucleatum* highlighted in recent work by Gallimidi et al. This study, using a novel mouse model for periodontitis-associated oral cancer has observed the synergistic effects of *P. gingivalis* with oral opportunistic pathogen, *F. nucleatum*, resulting in increased tumor severity, cell proliferation and inflammatory mediators via epithelial-expressed TLR2 activation (Binder Gallimidi et al., 2015). Our primary cell system demonstrated phenotypic results in a Scratch (Liang et al., 2007) and Zone of Exclusion Migration Assay (Holt and Cochrane, 2017) which show a slightly higher wound closure in *P. gingivalis* and *F. nucleatum* co-infected cells that could be biologically significant (Figures 6, 7). However, the migration promoted by *P. gingivalis* in primary GECs is significantly higher than that of *F. nucleatum* infection alone which suggests the early migration may be specific to *P. gingivalis*. This however, does not discount the morphological differences induced by *F. nucleatum* which may be significant in promoting the invasive phenotype. *P. gingivalis* infection also promotes the invasive capacity of immortalized epithelial cells and oral squamous cell carcinoma cells (Inaba et al., 2014; Ha et al., 2015; Sztukowska et al., 2016; Geng et al., 2017). However, in our primary OEC model, we did not anticipate an highly invasive phenotype as shown in other studies (Inaba et al., 2014; Ha et al., 2015; Sztukowska et al., 2016; Geng et al., 2017), since we are investigating initial molecular EMT changes in primary OECs that are not susceptible to metastatic transformation as immortalized cells are suggested to be (Maqsood et al., 2013; ATCC, 2016). Therefore, this study critically further validates some of the published literature but also provides additional physiologically invaluable information on initial EMT changes occurred during *P. gingivalis* infection in human primary OECS. Moreover, sustained levels of *P. gingivalis* survival in the host cells are highlighted and visualized in this study which has never been demonstrated before along with EMT molecules.

In summary, our study aimed to examine whether *P. gingivalis* infection is able to upregulate canonical EMT markers in primary human OECs and describe the responsible molecular events which *P. gingivalis* employs to trigger EMT signaling. The results suggest that long term *P. gingivalis* infection strongly associates with the induction of an early EMT process initiated by the phosphorylation of GSK3β in primary human OECs. This study for the first time demonstrates the EMT signaling in human primary OECs in the presence of *P. gingivalis* infection and provides timely novel information on the putative etiological role of *P. gingivalis* in the incidence and development of oral cancer. It is tempting to suggest that specific bacterial secreted effectors (e.g., Nucleoside-diphosphate-kinase) and/or structural virulence properties (e.g., Fimbriae or LPS) may contribute to the EMT independently of the presence of live intracellular organism in oral epithelial cells and future detail mechanistic studies are warranted to elucidate this complex EMT event in the context of host-pathogen interaction in oral mucosa.

## Ethics statement

Oral tissue that would otherwise be discarded was collected after informed consent was obtained by all patients under the approved guidance of the University of Florida Health Science Center Institutional Review Board (IRB, human subjects assurance number FWA 00005790). No human subject recruitment *per se* was done. Adult patients were selected at random and anonymously from those presenting at the University of Florida Dental Clinics for tooth crown lengthening or impacted third molar extraction. No patient information was collected.

## Author contributions

Conceived and designed the experiments: JL and ÖY. Performed and assisted the experiments: JL, JR, KA, NC, and KH. Analyzed the data: JL, JR, KH, and ÖY. Contributed reagents, materials, analysis tools: KH and ÖY. Wrote the paper: JL, JR, and ÖY.

### Conflict of interest statement

The authors declare that the research was conducted in the absence of any commercial or financial relationships that could be construed as a potential conflict of interest.
