# Supplementary material for: Human Primary Epithelial Cells Acquire an Epithelial-Mesenchymal-Transition Phenotype during Long-Term Infection by the Oral Opportunistic Pathogen, Porphyromonas gingivalis
Source: Front Cell Infect Microbiol. 2017 Dec 1;7:493. doi: 10.3389/fcimb.2017.00493 (PMC5717492; doi:10.3389/fcimb.2017.00493)
Supplement: Supplementary file 1 [file Presentation1.PDF]

Supplementary Figure Methods

MTT Assay (3-(4,5-Dimethyl-2-thiazolyl)-2,5-diphenyl-2H-tetrazolium bromide)

OECs were seeded in a 96-well plate and infected with *P. gingivalis* MOI 10 or MOI 100 for 120 hours. At the end of the infection time course, cell culture medium was replaced with Hank's Balance Salt Solution (HBSS) and 10 µl of MTT solution (12mM in PBS; Sigma) was added into each well and incubated for 4 h at 37^0C. The HBSS was then removed and 100 µl of DMSO was added into each well and the yellow-color MTT absorbance was detected at 540 nm with a Biotek H1M Microplate Reader (Biotek). Staurosporine [2µM] (Sigma) was used as a positive control for induction of apoptosis. Furthermore, DMSO concentrations ranging from 0.01%-0.1% were also added to the OECs to confirm no toxicity was caused in the OECs due to addition of pharmacological inhibitors containing DMSO.

Immunofluorescence

OECs were seeded on glass coverslips (Warner Instruments) in four-well plates (ThermoFisher Scientific) and infected at an MOI 100 with *P. gingivalis* 33277 for 120h. The staining protocol is explained in the Main Methods of the paper. Briefly, cells were immunostained with anti-*P. gingivalis* 33277 rabbit antibody followed by Alexa Fluor 488 goat anti-rabbit (Invitrogen) at 1:1000 dilution at ambient temperature for 1h. Cells were further incubated with rhodamine-phalloidin (Invitrogen) at 1:3000 dilution for 1h. The immunostained cells were mounted on glass slides using VectaShield mounting media with DAPI, and examined using wide-field fluorescence microscope (Zeiss Axio imager A1). The images were captured using a cooled charge-coupled device camera controlled by QCAPTURE software (Qimaging).

Antibiotic Protection Assay Quantified via 16sRNA

P. gingivalis survival in OECs was determined using an adapted approach from previously described antibiotic protection assays (Lamont et al. 1995; Choi et al. 2011). Briefly, OECs were infected with *P. gingivalis* in 6-well plates for 72, 96, and 120h under normal cell culture conditions. Cells were washed with PBS and remaining external bacteria were killed with gentamicin (300 µg/mL) and metronidazole (200 µg/mL) for 1h. GECs were then washed with PBS and Total RNA was isolated from *P. gingivalis* infected cells using 1mL Trizol Reagent (Invitrogen). Genomic DNA contamination was removed by DNase I (Ambion) digestion and samples were further purified using the RNeasy PLUS Mini Kit (Qiagen, Valencia, CA). cDNA was synthesized from 2 µg Total RNA using High Capacity cDNA Reverse Transcriptase Kit (Applied Biosystems). A 1:10 dilution of cDNA was used to detect *P. gingivalis* 16s RNA by SYBR Green Real-time quantitative PCR (Forward: 5′-TGTAGATGACTGATGGTGAAAACC-3′; Reverse: 5′-ACGTCATCCCCACCTTCCTC-3′) (Tran et al 1996). qPCR was carried out in CFX96 real-time system (Bio-Rad) with an initial cycle of 98°C for 3 min followed by 40 cycles of 95°C for 15s, 60.7°C for 30s, and 72°C for 30s. The CFU was calculated from the measured Ct (Threshold cycle) value of each infected-OEC condition by using a standard curve prepared with serial dilutions of genomic DNA of *P. gingivalis* of known CFU and their corresponding Ct values (Lyons et al 2000).

Supplementary Methods References

Lamont RJ, Chan A, Belton CM, Izutsu KT, Vasel D, and Weinberg A. Porphyromonas gingivalis invasion of gingival epithelial cells. Infection and Immunity, 1995. 63(10):3878-85.

Choi CH, DeGuzman JV, Lamont RJ, and Yilmaz Ö. Genetic transformation of an obligate anaerobe, P. gingivalis for FMN-green fluorescent protein expression in studying host-microbe interaction. PLoS One, 2011. 6(4):e18499.

Tran SD, and Rudney JD. Multiplex PCR using conserved and species-specific 16S rRNA gene primers for simultaneous detection of Actinobacillus actinomycetemcomitans and Porphyromonas gingivalis. J Clin Microbiol, 1996. 34(11): p. 2674-2678.

Lyons SR, Griffen AL, and Leys EJ. Quantitative real-time PCR for Porphyromonas gingivalis and total bacteria. J Clin Microbiol, 2000. 38(6): p. 2362-2365.

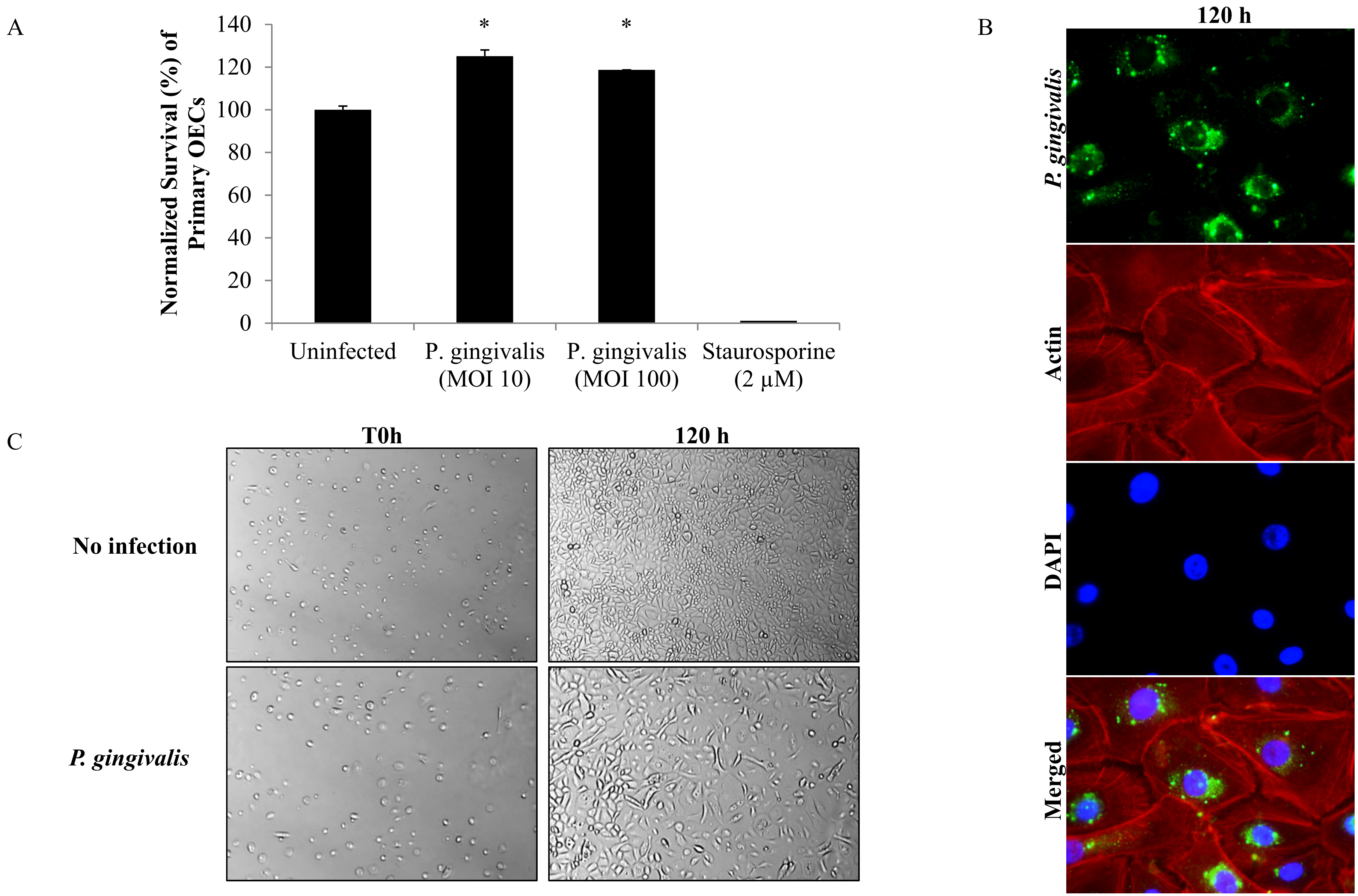

Supplementary Figure 1. OECs are viable during 120h long-term *P. gingivalis* infection. A. OECs were infected with *P. gingivalis* at MOI 10 or MOI 100 for 120h. Cell viability was assessed by the MTT assay. OECs were treated with staurosporine [2µM] as an inducer of apoptosis (positive control). DMSO did not have any effect on cell viability over 120h (not shown). The data is represented as mean +/-SEM normalized to Uninfected; n=6; *p<0.05. B. Fluorescent micrographs of OECs infected with *P. gingivalis* (green) for 120 hours and co-stained with actin (red) and DAPI (blue). Images were taken using Zeiss Axio Imager A1; 40x magnification. C. Bright-field micrographs of OECs prior to *P. gingivalis* infection and after 120 hours of infection shows OECs are attached and viable. Images were taken using Zeiss Invertoskop 40C; 5x magnification.

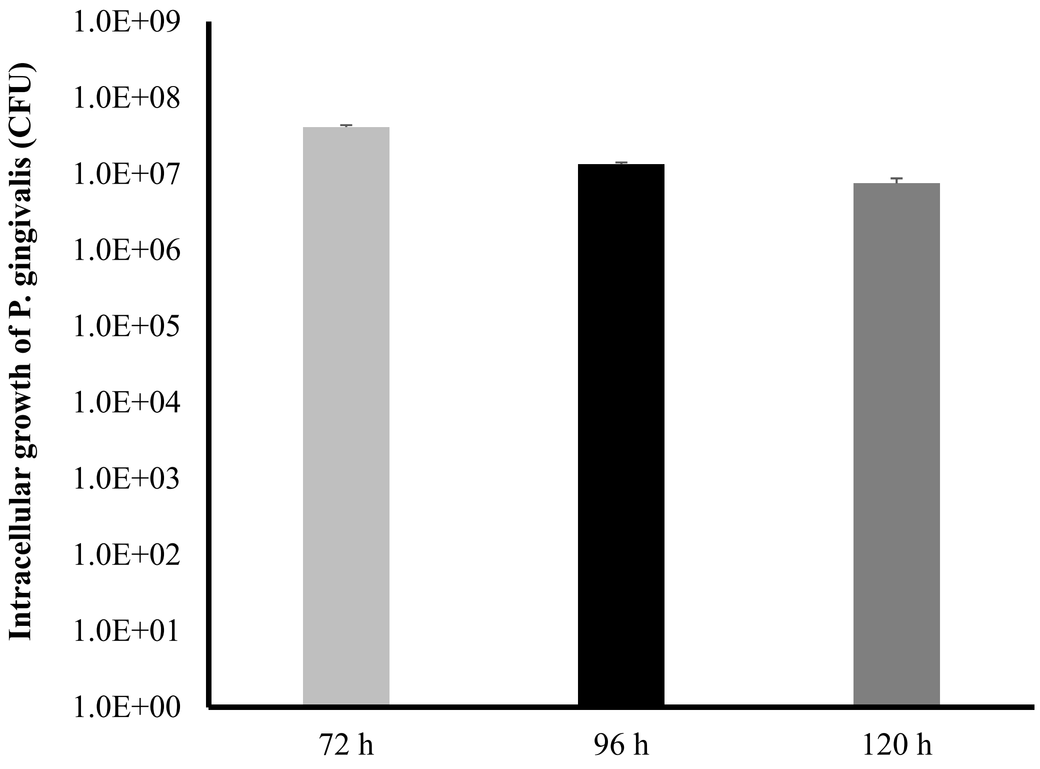

Supplementary Figure 2. ***P. gingivalis*** **is metabolically active in primary OECs over 120 hours of infection.** OECs were infected with *P. gingivalis* (MOI 100) for 72, 96, and 120h. Extracellular bacteria were killed by the addition of gentamicin (300 µg/mL) and metronidazole (200 µg/mL). Metabolically active *P. gingivalis* were determined by measurement of 16s RNA via qPCR. Colony-forming units (CFU) were then calculated from a previously determined standard curve (CFU versus *P. gingivalis* DNA Ct-values). The data is represented as mean +/-SEM; n=6.

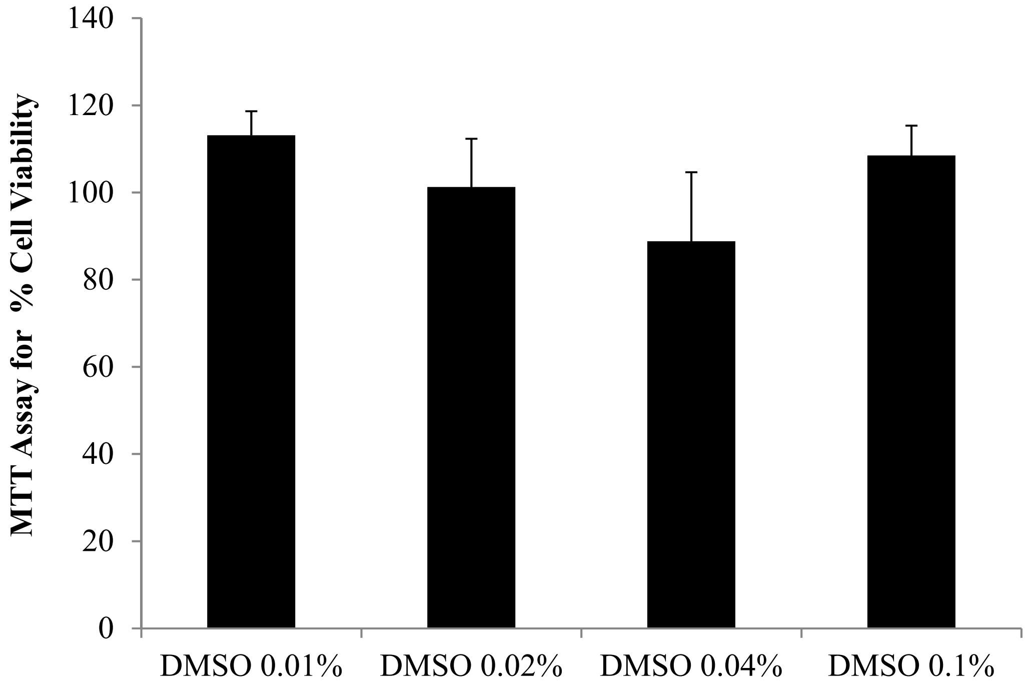

Supplementary Figure 3. DMSO concentrations (0.01-0.1%) do not negatively effect primary OEC Viability. OECs were treated with varied concentrations of DMSO for 24 hours. Cell viability was assessed by the MTT assay (3-(4,5-Dimethyl-2-thiazolyl)-2,5-diphenyl-2H-tetrazolium bromide). The data is represented as mean +/-SEM and normalized to Untreated; n=6. Changes were not found to be significant according to Students t-test.
